# Human Excreta as a Stable and Important Source of Atmospheric Ammonia in the Megacity of Shanghai

**DOI:** 10.1371/journal.pone.0144661

**Published:** 2015-12-14

**Authors:** Yunhua Chang, Congrui Deng, Anthony J. Dore, Guoshun Zhuang

**Affiliations:** 1 Center for Atmospheric Chemistry Study, Department of Environmental Science and Engineering, Fudan University, Shanghai, China; 2 Shanghai Key Laboratory of Atmospheric Particle Pollution and Prevention (LAP3), Department of Environmental Science and Engineering, Fudan University, Shanghai, China; 3 Centre for Ecology and Hydrology, Penicuik, Midlothian, United Kingdom; DOE Pacific Northwest National Laboratory, UNITED STATES

## Abstract

Although human excreta as a NH_3_ source has been recognized globally, this source has never been quantitatively determined in cities, hampering efforts to fully assess the causes of urban air pollution. In the present study, the exhausts of 15 ceiling ducts from collecting septic tanks in 13 buildings with 6 function types were selected to quantify NH_3_ emission rates in the megacity of Shanghai. As a comparison, the ambient NH_3_ concentrations across Shanghai were also measured at 13 atmospheric monitoring sites. The concentrations of NH_3_ in the ceiling ducts ($2809_{-2661}^{+5803}$ μg m^-3^) outweigh those of the open air (~10 μg m^-3^) by 2–3 orders of magnitude, and there is no significant difference between different seasons. δ^15^N values of NH_3_ emitted from two ceiling ducts are also seasonally consistent, suggesting that human excreta may be a stable source of NH_3_ in urban areas. The NH_3_ concentration levels were variable and dependent on the different building types and the level of human activity. NH_3_ emission rates of the six residential buildings (RB_NH3_) were in agreement with each other. Taking occupation time into account, we confined the range of the average NH_3_ emission factor for human excreta to be 2–4 times (with the best estimate of 3 times) of the averaged RB_NH3_ of 66.0±58.9 g NH_3_ capita^-1^ yr^-1^. With this emission factor, the population of ~21 million people living in the urban areas of Shanghai annually emitted approximately 1386 Mg NH_3_, which corresponds to over 11.4% of the total NH_3_ emissions in the Shanghai urban areas. The spatial distribution of NH_3_ emissions from human excreta based on population data was calculated for the city of Shanghai at a high-resolution (100×100 m). Our results demonstrate that human excreta should be included in official ammonia emission inventories.

## Introduction

Ammonia (NH_3_) is the principal alkaline gas capable of neutralizing the acid species (e.g., H_2_SO_4_, HNO_3_) produced by the oxidation of SO_2_ and NO_2_ in the atmosphere, to form ammonium nitrate (NH_4_NO_3_) and ammonium sulfates (NH_4_HSO_4_ and [NH_4_]_2_SO_4_) [1]. These soluble ammonium aerosol salts are important components of airborne fine particulate matter (PM_2.5_) that, as stressors, can significantly contribute to regional haze [2, 3] and subsequently affect human health [3, 4]. The importance of NH_3_ as an environmental stressor in China is expected to increase because most of the regulatory efforts have been directed towards SO_2_ and NO_x_ reductions [4–6]. However, following the lead of the European Union, China introduced the central government's first-ever technical guideline for an NH_3_ emission inventory in August 2014 [7]. This will help quantify the contribution of major national emissions sources contribution to high levels of particulate matter formation.

Over recent decades, the release of NH_3_ into the atmosphere in many regions has been substantially modified by human activities [8–11]. Of the many natural and anthropogenic sources, emissions from the microbial breakdown of urea and uric acid in animal excreta make the largest contribution globally and regionally [12–14]. The amount of NH_3_ volatilized from bacterial decomposition is dependent on many factors, including in decreasing order of importance: litter pH, temperature, and moisture content. In the “bottom-up” methodology, NH_3_ emissions are calculated as a product of the emission coefficients and activity level (e.g., livestock population, fertilizer consumption and production). Improved estimates of NH_3_ emission factors (EF) are therefore central to reducing the uncertainties.

As a normal metabolic process, the release of NH_3_ from human, respiration and excrement has also been documented [11, 15, 16]. However, most emission inventories involving human excreta have merely focused on pit latrines in rural areas of developing and middle-income countries [17, 18]. The traditional view maintains that emissions in urban areas can be omitted since excrement of N from humans immediately enters the sewage system by virtue of flush toilets [18]. This may be true that in the EU and US, where human excreta along with wastewater are directly transported through sewage pipes to sewage treatment plants [19], and the whole process will not lead to any considerable losses of NH_3_ to the atmosphere [20].

While in urban China, human excreta are typically stored in a three-grille septic tank under the building at first. After a series of anaerobic decomposition processes, a substantial amount of odors (including NH_3_) will be generated and emitted through a ceiling duct. The liquid supernatant will then flow away from the third grille to the municipal sewage system. Municipal sanitation workers will routinely dredge the tank typically once a year. As China’s urbanization is expanding rapidly, the contribution of on-site septic systems to NH_3_ might be highly significant at a local scale (e.g., urban areas). More background information regarding human excreta in China and Shanghai is available in the supporting information (S1 Text).

After a critical evaluation of existing literature, we noticed that in contrast to the detailed information available on NH_3_ EFs for animal manure and fertilizer application, data on the source strength of human emissions has not been quantitatively determined. For human sweat, Healy et al. [21] gave an emission coefficient of 260 g NH_3_-N capita^-1^ yr^-1^ by assuming 5% of total N excretion (30 g day^-1^ capita^-1^) occurring through sweat were completely hydrolyzed and volatilized. Similarly, Buijsman et al. [22] introduced an ammonia EF for human respiration as 300 g NH_3_ capita^-1^ yr^-1^ without detailed explanation. As to human excrement, it is surprising to find the earliest and only EF suggested for humans excreta (1300 g NH_3_-N capita^-1^ yr^-1^, including excrement, sweat and respiration) is that of Möller and Schieferdecker [23], who gave an N production of around 5000 g N capita^-1^ yr^-1^; simply assuming a NH_3_ loss of 25%. Based on those parameters, various NH_3_ EFs associated with humans were proposed and then applied to the estimate of NH_3_ emission inventories. To some extent, existing theoretical EFs can be accepted in rural areas of developing and middle-income countries, where there is a lack of basic sanitation. Experimentally, however, study of the EF of NH_3_ from human excreta in urban areas has been missing.

The primary objective of this study is to quantify the EF and emission inventory of NH_3_ emissions from human excreta in urban Shanghai, proving that human excreta is a missing source of NH_3_. Intensive field monitoring campaigns were performed involving measurement both in and out of ceiling ducts to obtain first-hand information about the concentration level, emission rate, and isotopic composition of NH_3_ emitted from human excreta across Shanghai.

## Materials and Methods

### 1. Building selection

Thirteen buildings with six functions (six residential buildings, one hotel, two office buildings, one teaching building, two student apartments, and one community culture center) were chosen for sampling either because they are common in Shanghai (e.g., residential building) or could be implemented as improved treatment technologies for human excreta (e.g., office building). All the sampling work in the current study was conducted with the permission of these building owners (S1 Table), without any endangered species involved. Human excreta produced in each building were flushed into a septic tank beneath the building. The last dredge removal of all septic tanks was at least six months previously, and all excess gaseous NH_3_ generated from the septic tanks must be emitted to the open atmosphere via PVC (polyvinyl chloride) pipes. These buildings are scattered over seven major urban districts of Shanghai to represent the entire city. S1 Table details the information of the buildings and the socio-economic status of the occupants.

**Fig 1:**
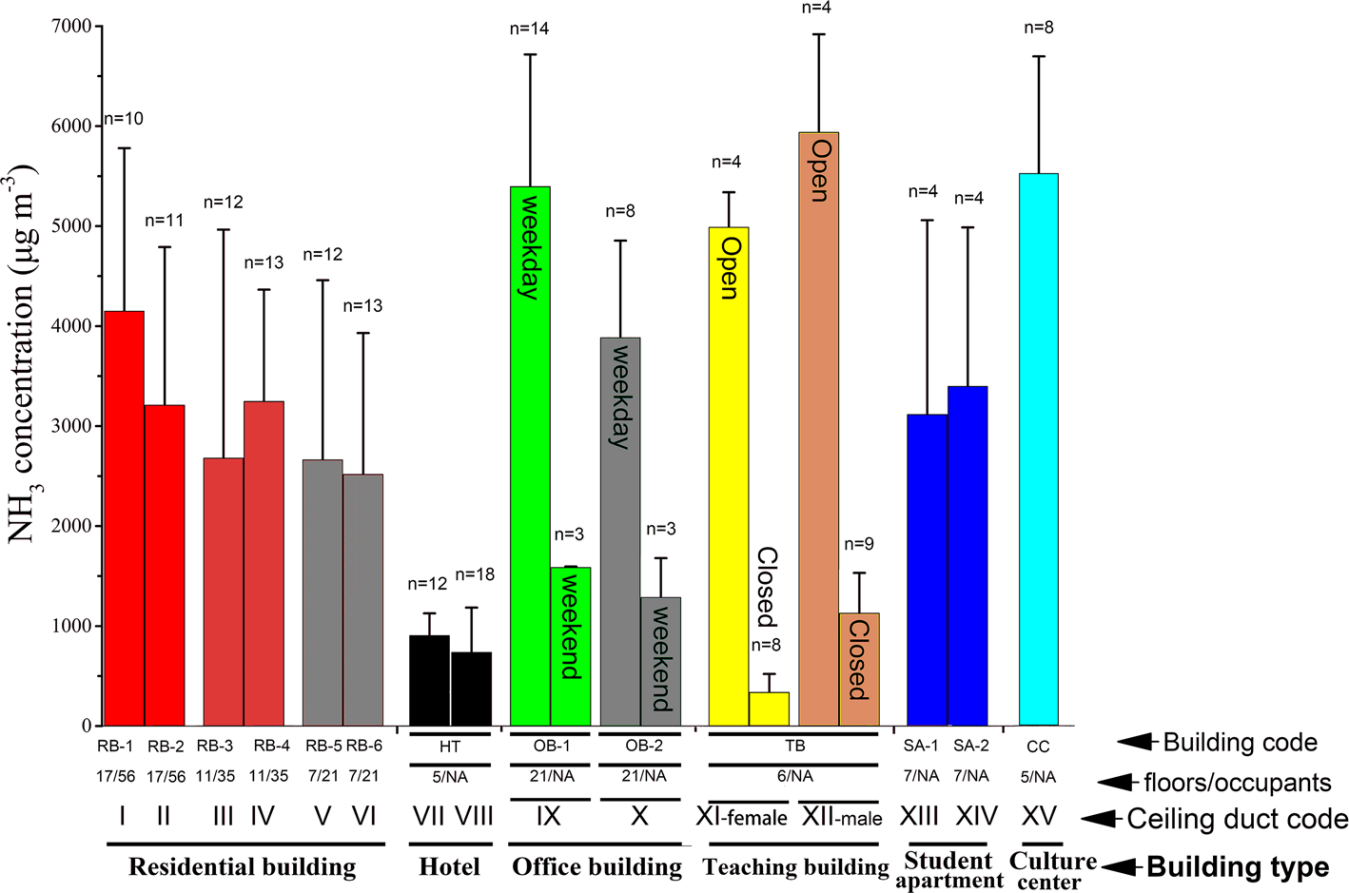
Comparison of NH_3_ concentrations collected from different ceiling ducts among different buildings. Each building or ceiling duct has a unique code or Roman numeral (see the text of the *building selection* section and S1 Table for detailed information). Floors/occupants represent the number of floors/occupants in each building, and NA means an accurate number is not available. For office and teaching buildings, there is also a comparison of NH_3_ concentrations collected from the same ceiling duct of a building between different periods (e.g., weekday/weekend, school day (open)/non-school day (closed)).

Each building unit has one septic tank, most of which have a 12-m^3^ standard volume (except for the septic tanks of office buildings with 60-m^3^ volume) and are connected to the atmosphere with one ceiling duct. It should be noted that the teaching building has two ceiling ducts for male and female toilets, respectively. Although there were four ceiling ducts in the rooftop of the Yandang Hotel, two of them were surrounded by solar panels for water heating, and the property service of the hotel refused our request of sampling in these two ceiling ducts. These four ceiling ducts shared the same septic tank. Therefore, there are 13 buildings, 13 septic tanks and 15 ceiling ducts in the current study. For demonstration purposes, each building (ceiling duct) is denoted by a code (Roman numeral) (Fig 1). Specifically, the codes of the six residential buildings (residential buildings’ six ceiling ducts) are RB-1 (I), RB-2 (II), RB-3 (III), RB-4 (IV), RB-5 (V), and RB-6 (VI), respectively. The code of the hotel (hotel’s two ceiling ducts) is HT (are VII and VIII). The codes of the two office buildings (office building’ two ceiling ducts) are OB-1 and OB HT (IX and X). The code of the teaching building (teaching building’s two ceiling ducts) is HT (are XI-female and XII-male). The codes of the two student apartments (student apartments’ two ceiling ducts) are SA-1 and SA-2 (XIII and XIV). The code of the community culture center (community culture center’s ceiling duct) is CC (XV).

It is worth noting that to obtain the exact number of the occupants in a specific building was not a simple calculation. It was also very difficult to know the time they spend in their home. However, this information was easier to ascertain for residential buildings and student apartments since those data can be provided by the property services. Given its overwhelming dominance and relatively sound data availability, the NH_3_ EFs of human excreta in the current work were based on the emission rates of the six residential buildings.

### 2. Sampling and NH_3_ concentration determination

Ambient NH_3_ measurements in the current study were collected using Ogawa Passive Sampling Devices (Ogawa & Co., Inc., Pompano Beach, Florida), a cost-effective tool that has been widely used to determine the time-averaged NH_3_ concentrations [24, 25]. The Ogawa PSD is composed of a solid cylindrical polymeric body with two reactive glass-filters on each side impregnated with citric acid. The filters were purchased from the manufacturer. The NH_3_-containing air mass originating from human excreta was conducive to measure in a building equipped with a septic tank, where sampling devices can be located in the interior of the ceiling duct, the only opening to the atmosphere. To prevent potential perturbation of the outdoor environment, measurements were taken approximately 1.5 m from the exhaust end of the ceiling duct. Duplicate 24-h samples (normally from 12:00 to 12:00 of the next day) of NH_3_ concentrations were non-consecutively collected from the interior ceiling duct of the selected buildings in Shanghai urban areas, during the period from July to August of 2014, and December 2014 to March 2015. Sampling in wintertime was performed at the teaching building (ceiling duct of XI-female and XII-male), two students apartments (ceiling duct of XIII and XIV), and a residential building (building code of RB-5, ceiling duct of V). Interior temperature and wind speed were simultaneously detected by a probe linked to the wind data logger (WFWZY-1, Tianjian Huayi, Beijing, China), which was next to the sampler and recorded every three seconds or one minute during sampling. Additionally, two field blank samples for each site were also collected and analyzed to determine if contamination occurred during the sampler loading, transport, or analysis. All field blanks were well below sample concentrations, mostly were zero. The wind speed and NH_3_ concentrations in the air at the point of measurement, two key parameters to estimate emission rate, were assumed to be the same as at the end of the exhaust. A schematic of the sampling is shown in S1 Fig.

Weekly concentrations of NH_3_ at thirteen atmospheric monitoring sites that covers various locations including rural (Dianshanhu), remote island (Huaniao), urban (Fudan, Hongkou, Yangpu, Huangpu, Putuo, Jing’an, Xuhui, Luwan, Pudong), and suburban (Chuansha and Zhangjiang) areas were also measured by the Ogawa PSD, in which all ten state controlling sites in Shanghai were involved. In addition, hourly concentrations of NH_3_ at the Pudong air quality research supersite in the center of Shanghai (121.54^0^E, 31.23^0^N) were also measured in parallel by a Monitor for AeRosols and GAses system (MARGA, ADI 2080 1S, Metrohm AG, the Netherlands) [26] for inter-comparison with the passive sampler from June to December 2014. S2 Fig illustrates a significant correlation (N = 31, R^2^ = 0.63, slope = 0.79, P<0.001) between the two measurement methods, verifying the validity and reliability of the Ogawa passive sampler we used. The sampling, operation and internal calibration of the MARGA were in strict accordance with the instructions provided by the manufacturer.

Filter samples were sealed and stored in the refrigerator (4°C) before analysis. In the laboratory, samples were analyzed following the manufacturer’s protocols (http://www.ogawausa.com), firstly soaked with ultra-pure water (18.2 MΩ.cm) for 30 min, shaking occasionally. Concentrations of NH_4_^+^ were then analyzed using an ion chromatographic system (883 Basic IC plus, Metrohm Co., Switzerland) equipped with a Metrosept C4/4.0 cation column. The eluent was 1.0 mM HNO_3_ + 0.5 mM PDA. The detection limit for NH_4_^+^ was 2.8 μg L^-1^. The analytical errors in the measurements of NH_4_^+^ during duplicate analyses of laboratory standards were within 5%. The concentrations (in μg m^-3^) reported here were corrected for the corresponding field blanks and converted to dry standard conditions (21.1°C and 101.3 kPa).

### 3. Estimation of NH_3_ emission factor, emission inventory and its spatial allocation

An emission rate is an expression of mass emitted per unit time, typically in units of ‘g day^-1^’. In our study, the ceiling duct can be viewed as a one-way outflow channel of NH_3_ emission (from the septic tank to the open atmosphere through a PVC pipe). Therefore, a flow-based method of emission rate calculation is used in the current study. Specifically, the NH_3_ emission rate of human excreta from a septic tank for a selected building is the function of air flux (wind speed), sampling time, and NH_3_ concentrations in its ceiling duct:

$$\begin{array}{l} {\text{ER}}_{i•j}=\pi \times r^{2}\times \left(86400\times {\text{W}}_{i•j}\right)\left({\text{C}}_{i•j}/1000000\right) \end{array}$$ (1)

Where ER is the emission rate (g day^-1^); *i∙j* is ceiling duct *i* in building *j*; r is the radius of ceiling duct (0.11 m); 86400 is the number of seconds in a day; W is the daily average wind speed (m s^-1^); and C is the measured daily concentration of NH_3_ (μg m^-3^); and 1000000 is the coefficient of mass concentration unit conversion from μg to g. The distribution of NH_3_ emission from human excreta in urban Shanghai was spatially allocated to population density, which was depicted by the mapping software of a geographic information system (GIS) (ArcGIS 10.2, ESRI, Redlands, California). In our work, the MODIS global map of urban distribution with a spatial resolution of 250×250 m was utilized to separate a newly released high-resolution (100 m^2^ per cell) population map (http://www.worldpop.org.uk/). The roads network, administrative boundaries with a scale of 1:250 000 were extracted from the National Geometrics Center of China (http://ngcc.sbsm.gov.cn/).

### 4. Isotopic Analysis

Recently, a novel and robust chemical method for δ^15^N-NH_4_^+^ at natural abundance has been developed and used in the current study. The detailed analytical procedures are given elsewhere [27]. Briefly, this method is based on the isotopic analysis of nitrous oxide (N_2_O), which is much less abundant in the atmosphere than N_2_ and thus causes minimal atmospheric contamination. Eight samples collected in summer and winter were chosen for isotopic analysis, four of them were collected from the ceiling duct of XII (13 July, 5 August of 2014; 7 January, 14 January of 2015) and the other four were collected from the ceiling duct of V (16 July, 7 August of 2014; 9 January, 12 January of 2015). After measurement by IC as stated above, NH_4_^+^ in the aqueous sample was initially oxidized to nitrite (NO_2_^-^) by hypobromite (BrO^-^) in a vial. NO_2_^-^ was then quantitatively converted into N_2_O by hydroxylamine (NH_2_OH) under strongly acid conditions. The produced N_2_O was analyzed by a purge and cryogenic trap system (Gilson GX-271, IsoPrime Ltd., Cheadle Hulme, UK) coupled to an IRMS (PT-IRMS) (IsoPrime 100, IsoPrime Ltd., Cheadle Hulme, UK) at the Stable Isotope Ecology Laboratory of Institute of Applied Ecology, Chinese Academy of Sciences.

Isotope ratio values are reported in parts per thousand relative to atmospheric N_2_ as follows:

δN15(‰)=(15N/14N)sample-(15N/14N)N2(15N/14N)N2×1000 (2)

Three international NH_4_^+^ standards (IAEA N1, USGS 25, and USGS26 with δ^15^N values of +0.4‰, -30.4‰ and +53.7‰, respectively) were used to correct for the reagent blank and drift during isotope analysis of the produced N_2_O. The standard deviation of δ^15^N measurements is less than 0.3‰ [27].

## Results

In total 170 samples collected from the 15 ceiling ducts in summertime were analyzed. The overall concentrations of NH_3_ were highly variable and ranged from 148–8612 μg m^-3^, with an average value (±standard deviation) and median value of 2809 (±2032) and 2078 μg m^-3^, respectively (Fig 1). One thing which should be noted is that the minimum value was found at ceiling duct XI when the teaching building was closed due to the summer vacation (Fig 1).

Specifically, NH_3_ concentrations in the ceiling ducts of the teaching building (XI-female and XII-male), the office buildings (IX and X) and the community culture center (XV) were among the highest, in agreement with our direct observation that those places were always fully occupied by people. The hotel we chose is of a typical middle-size (5 floors with 35 rooms in total) with two more ceiling ducts apart from VII (905±222 μg m^-3^) and VIII (738±448 μg m^-3^); therefore, the NH_3_ emissions of the entire building could be much greater than the current estimate. For the teaching building, two ceiling ducts (XI-female and XII-male) share one septic tank. The samples collected during summer vacation (337±184 μg m^-3^ for XI-female, and 1128±404 μg m^-3^ for XII-male) were significantly lower (P<0.001) than during a normal teaching semester (4988±351 μg m^-3^ for XI-female, and 5937±982 μg m^-3^ for XII-male). The higher NH_3_ concentrations in XII-male than that of XI-female could be explained by the intense renovation work by male workers during the 2014 summer vacation. Weekend samples of NH_3_ for the two office buildings (1585±11 μg m^-3^ for IX, and 1286±393 μg m^-3^ for X), similarly, were lower than the samples collected during weekdays (5395±1322 μg m^-3^ for IX, and 3884±971 μg m^-3^ for X) but with moderate declining rates. This is because many language training programs were arranged at weekends in the two buildings, while the residential buildings and student apartments have no obvious weekend effect. We can conclude that NH_3_ emitted from the ceiling ducts of various buildings are ubiquitous in Shanghai, and their concentration levels are closely related to the strength of human activity.

In Table 1, the NH_3_ concentrations emitted from the ceiling ducts of V, VII, VIII and XIV in winter are close to summer, suggesting that there is no seasonal effect on the NH_3_ emissions from urban *in situ* septic tank system.

**Table 1:** Seasonal comparison of NH_3_ concentrations and *δ* ^15^N-NH_3_ values collected from different buildings.

		Summer			Winter		
Building	Ceiling duct	n	T (°C)	NH_3_ (μg m^-3^)	n	T (°C)	NH_3_ (μg m^-3^)
RB-5	V	12	28.3 mg	2662 mg d	4	7.82 mg	3126 mg d
TB	XII	4^a^	27.7 mg	5937 mg	4^a^	7.67 mg	5554 mg d
SA-1	XIII	4	27.9 mg	3116 mg d	4	9.16 mg	2980 mg d
SA-2	XIV	4	27.8 mg	3397 mg d	4	9.57 mg	3174 mg d
Building	Ceiling duct	n	T (°C)	δ^15^N (‰)	n	T (°C)	δ^15^N (‰)
RB-5	V	2	28.8))g	-39.3, -38.1	2	7.9.3,	-37.9, -39.2
TB	XII	2	28.39, -	-37.3, -39.0	2	7.5.3,	-37.4, -39.6

a: samples collected during weekdays

## Discussion

### 1. Unexpectedly high NH_3_ concentrations from human excreta

This study is the first attempt to quantify the concentrations of NH_3_ emitted from human excreta in ceiling ducts; consequently no other comparable published data is available at present [28]. The ambient levels of atmospheric NH_3_ over Shanghai, in stark contrast to the ceiling ducts, were several orders of magnitude lower, with concentrations ranging from <10 μg m^-3^ in coastal (Huaniao Island) and suburban sites (Chuansha and Zhangjiang) to 10–15 μg m^-3^ in urban (Huangpu, Luwan *et al*.) and rural sites (Dianshanhu). It is worth noting that during our study period, the weekly average concentrations of NH_3_ at two of the eight urban sites (Huangpu and Luwan) with high population density were constantly greater than that of the rural site (i.e., Dianshanhu) (discussed in the later section). Given the short atmospheric lifetime and short-range transportation of NH_3_, our monitoring results differ from previous emission inventories in highlighting the strong emissions of non-agricultural NH_3_ sources from local human activities (including human excreta) in urban areas. In conclusion, our results clearly show that human excreta is an important source of NH_3_ in Shanghai that deserves more attention.

Spanning 21 floors with nearly 24000 m^2^ of office space, each of the twin office buildings we chose can accommodate 2400 people for work. Unexpectedly, the NH_3_ concentrations of IX and X were not proportionally higher than other buildings with fewer occupants. This could be attributed to the application of activated carbon adsorption (ACD) system deployed in the septic tank. The ACD system was designed for new office buildings in Shanghai to remove odors, not specifically for NH_3_. Nevertheless, it has been shown in our study that such a system is highly efficient in term of NH_3_ sequestration. Gathering evidence supports our observation that an activated carbon modified by acid has greater potential for NH_3_ absorption [29, 30]. However, the application of the ACD system is still extremely rare in Shanghai at present (personal communication with the office building manager in charge of heating ventilation/air conditioning).

### 2. Human excreta as a stable NH_3_ source in Shanghai

Here the δ^15^N values of NH_3_ emitted from two ceiling ducts are also seasonally consistent (Table 1), suggesting that human excreta may be a stable source of NH_3_ in urban areas. Various NH_3_ sources have been reported to have distinctly different N isotopic compositions [31]. As the first attempt to determine the δ^15^N signature of NH_3_ emitted from human excreta, our results are much lower than that of fossil fuel combustion processes, like vehicles (-4.6‰ and -2.2‰) and coal combustion (-14.6‰ and -11.3‰), but are close to that of volatilization process, like cow waste (-27.1‰ and -23.1‰) [31]. Therefore, our results provide strong evidence that NH_3_ emitted from ceiling ducts are derived from human excreta.

### 3. Human excreta as an important NH_3_ source in Shanghai

**Fig 2:**
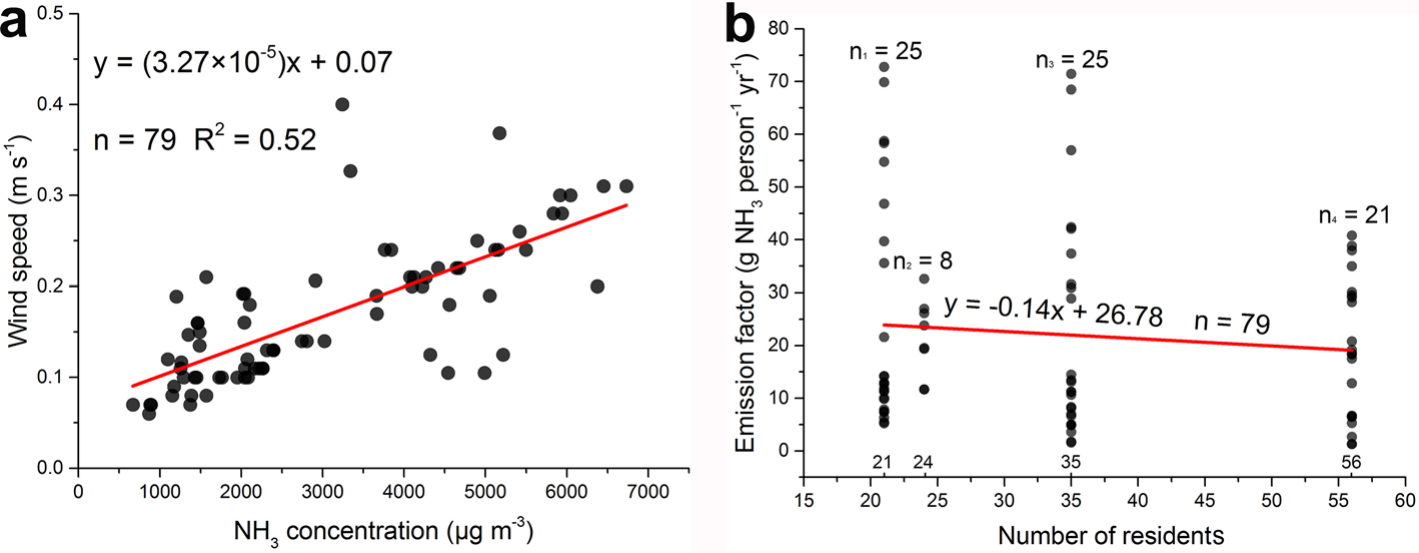
(a) Correlation between the NH_3_ concentration and the wind speed (air outflow velocity from septic tank to the open atmosphere through ceiling duct) for the 79 samples collected from the six residential buildings and two student apartments; (b) the 79 individual EFs are divided into four groups based on the number of occupants (x axis). A linear analysis is conducted to examine if different groups (the number of residents) have the same average EF.

Calculating from an emission rate, an EF is expressed in terms of mass per production unit. In this study, the production unit obviously refers to the number of occupants, and the NH_3_ EF from human excreta for a specific building is defined as annual total NH_3_ emitted per capita (g capita^-1^ yr^-1^). The six residential buildings and two student apartments are discussed together to estimate the NH_3_ EF because they have reliable information and similar building structures, allowing the possibility of mutual calibration. The measured NH_3_ concentrations and wind speeds from all residential buildings and student apartments are significantly correlated (R^2^ = 0.52, P<0.001), which confirms that wind speed in the ceiling duct is a good indicator of NH_3_ emission rate (Fig 2A).

**Fig 3:**
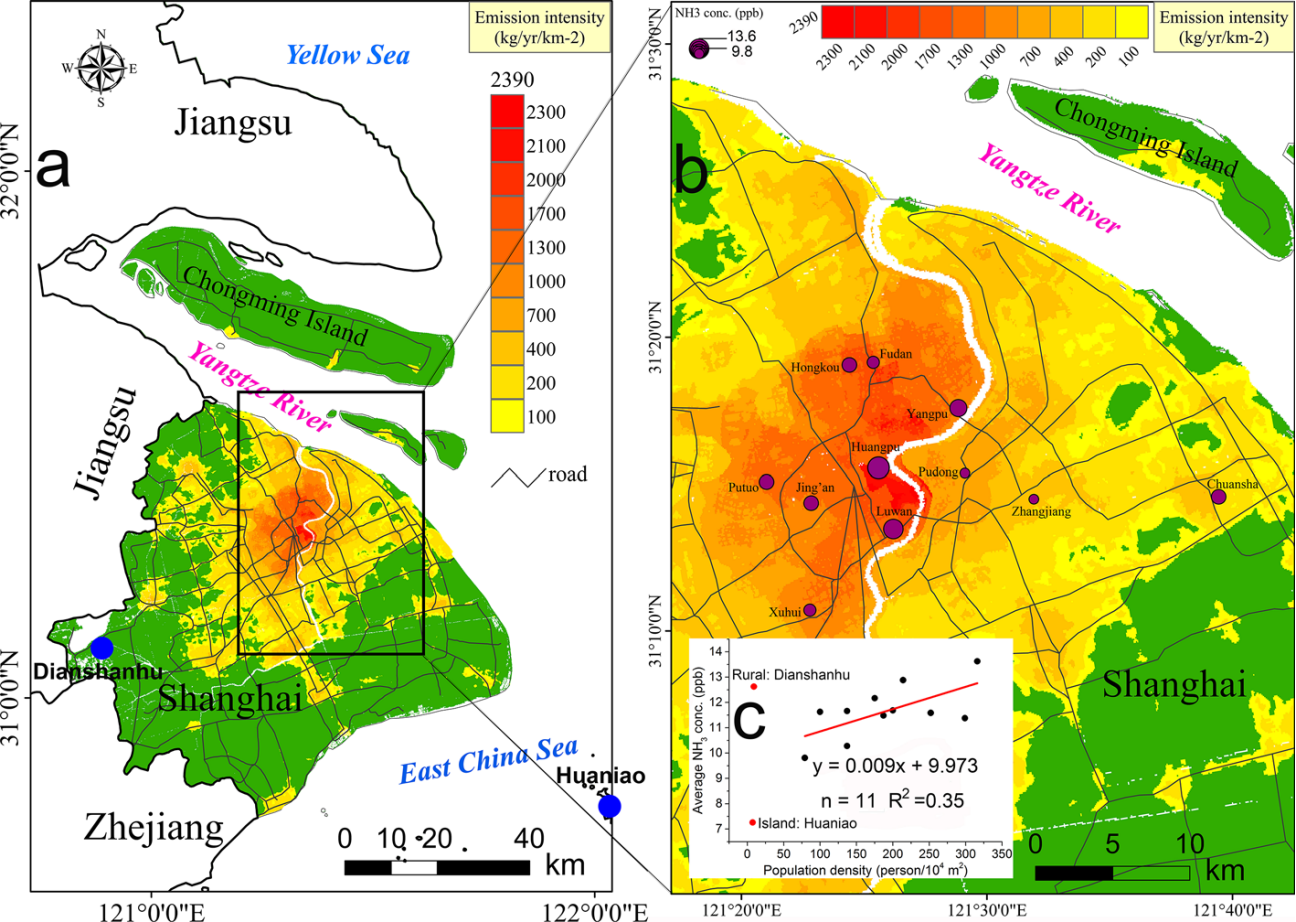
Spatial distribution of NH_3_ emissions from human excreta in Shanghai urban areas (areas in green represent the rural areas of Shanghai, which are out of consideration in the current work) (a). A close look at the distribution of nine urban sites (Fudan, Huangpu, Yangpu, Hongkou, Jing’an, Putuo, Xuhui, Luwan, and Pudong) and three suburban sites (Zhangjiang and Chuansha) and their NH_3_ monitoring results (depicted by the size of the circle) (b). Correlation analysis is conducted between the population density of each site (excluding Dianshanhu site and Huaniao site, marked in red) and their ambient NH_3_ concentration (c).

In our study, an assumption is implicit in the definition of EF: all residents in their own apartment share the same EF regardless of gender, age or any other kind of socioeconomic status. Although the production cycle for NH_3_ emissions from human excreta is of roughly one day, the whole production process is far from being an all-in all-out production system since humans frequently move from one building to another. Therefore, a time factor must be assigned to the EFs of residential buildings, which are the most typical and common building type in practice. Based on our visits and interviews with the occupants, we summarized an average time of people stayed in their apartment was 12-h a day, of which at least 6-h. were spent for sleep. Therefore, a reasonable NH_3_ emission for one person across a whole day could be 2 to 4 times of emissions in his/her apartment. There are 79 samples collected from the six residential buildings (RB-1 to RB-6) and two student apartments (SA-1 and SA-2). This represents 79 individual EFs. In our work, RB-1 and RB-2, RB-3 and RB-4, RB-5 and RB-6, SA-1 and SA-2 have the same number of occupants. Therefore, the 79 EFs can be divided into four groups based on the number of the occupants, i.e., 56 (RB-1 and RB-2), 35 (RB-3 and RB-4), 24 (RB-5 and RB-6), and 21 (SA-1 and SA-2). In Fig 3B, a linear analysis is conducted to examine if different groups have the same average EF. It is clear that the EFs among different groups present a slight downward trend (slope = -0.14), indicating that the averaged EFs of each group are in good agreement with each other and can be extrapolated to other residential buildings.

Based on the methodology and discussion above, the annual average EF of NH_3_ from human excreta (mean±1σ) for the six residential buildings and two student apartments is estimated as 22.0±19.4 g capita^-1^ yr^-1^ (RB_NH3_) and 21.5±7.4 g capita^-1^ yr^-1^ (SA_NH3_), respectively (see S2 Table for details). From a day-cycle perspective as discussed above, the NH_3_ EF for human excreta (HM_NH3_) should be 2×RB_NH3_ to 4×RB_NH3_, with a best estimate of 3×RB_NH3_, namely 66.0±58.9 g capita^-1^ yr^-1^. Sutton et al. [32] suggested a value of 13.7 (2.8–63.2) g NH_3_-N capita^-1^ yr^-1^ as the EF for an infant. However, there appears to be no HM_NH3_ in the literature that is determined by experiment. Here we call for a greater scientific effort to be allocated to this area of research. Based on the EF we calculated, the population of an estimated 21 million people living in Shanghai urban areas emitted approximately 1386±1222 Mg NH_3_ a year. According to Huang et al.[14] and Fu et al. [33], the total NH_3_ emissions over Shanghai were estimated as 42600 Mg (-36% to 77% uncertainty at the 95% confidence interval, similarly hereinafter) in 2007 and 64500 Mg (±112.8%) in 2010, which both made a significant underestimation of the NH_3_ emissions from urban areas. When compared with the NH_3_ emissions from Shanghai city area (10742 Mg) [34], the average contribution of our estimate can reach 11.4%. Moreover, model results have shown that over half of agricultural NH_3_ emissions would be deposited downwind of its source within 10 km depending on local meteorological conditions [35]. Therefore, the relative contribution of NH_3_ emissions from human excreta to urban PM pollution could be higher than the share of its mass contribution.

### 4. Spatial pattern of NH_3_ emissions from human excreta across Shanghai urban areas

Based on the population distribution [36] and the HM_NH3_ we developed, a panoramic view of NH_3_ emissions from human excreta in urban Shanghai is illustrated in Fig 3A. The emission intensity ranged from 132 kg km^-2^ to 2390 kg km^-2^, with an average value in the major urban areas of 1110 kg km^-2^. Those densely-distributed urban and suburban sites and their atmospheric NH_3_ monitoring results are clearly visible in Fig 3B. In Fig 3C, the NH_3_ mixing ratios from monitoring data at urban and suburban sites are shown to be significantly correlated with population density. The levels of NH_3_ concentrations at Huangpu (13.6 ppb) and Luwan (12.9 ppb) are the highest because they are located in regions of high population density. However, this principle cannot be applied to the rural site (Dianshanhu) and the coastal island site (Huaniao) in our study (Fig 3C). Huaniao Island can be regarded as a background site due to its sparse population. However, its ambient NH_3_ mixing ratio (7.3±1.7 ppb) is still higher than some big cities, like Houston (3.0±2.5 ppb) [37]. Riddick et al. [38] found that the excreta of seabirds at their breeding colonies might act as a notable source of NH_3_ emissions to the coastal atmosphere. For Dianshanhu, a good explanation for its high NH_3_ concentration might be the prevalence of agricultural activity, in particular urban agricultural production with high nitrogen fertilizer application. Agriculture is widely accepted to make the dominant contribution to atmospheric concentrations of NH_3_ However our measurements show that the ambient levels of NH_3_ concentration in Shanghai urban areas were comparable with, or even higher than those from Dianshanhu. Our current study on NH_3_ emissions from human excreta suggests that a wider discussion on the important role of non-agricultural NH_3_ emissions at a city scale is needed.

## Conclusions

Taking Shanghai as an example, the results of this study clearly reveal that human excreta is a stable and important NH_3_ source which should not be neglected in Shanghai urban areas. Shanghai is however by no means unusual in today’s China: Emerging from an economy dominated by agriculture, by the end of 2012, China has 656 cities and a total urban population of 712 million (52.6% of the total population), up from 69 in 1947 and 223 in 1980 [39]. Substantial changes in the size and spatial distribution of population are also expected in many other parts of the world this century. Therefore, the contribution of human excreta to ambient NH_3_ levels in cities is expected to increase steadily.

It is well known that China is now experiencing the world’s worst air quality in terms of PM_2.5_ pollution [3, 40]. As one of the key precursors of PM, the implications of limiting NH_3_ emissions to curb China’s deteriorating air quality would be enormous. The observation of unexpectedly lower NH_3_ emissions from the septic tank equipped with active carbon system suggests that these strong NH_3_ emissions from human excreta in urban areas could be effectively controlled by introducing a suitable sewage management policy. We expect our current work could inspire further research to develop a cost-effective way of reducing NH_3_ emissions from septic tanks in the future.

## Supporting Information

S1 Fig A schematic of the sampling in the exhaust of ceiling duct. (DOCX)

S2 Fig Comparison of NH_3_ concentration results obtained with the Ogawa passive sampler device and an active monitor (MARGA) at Pudong supersite from June to December, 2014. (DOCX)

S1 Table Information about the thirteen buildings with six function types. (DOCX)

S2 Table The NH_3_ concentrations, wind speed and emission factors of the six residential buildings and two student apartments. (DOCX)

S1 Text The background information of the production and storage of human excreta in Shanghai and China. (DOCX)
