# Supplementary material for: Human Excreta as a Stable and Important Source of Atmospheric Ammonia in the Megacity of Shanghai
Source: PLoS One. 2015 Dec 14;10(12):e0144661. doi: 10.1371/journal.pone.0144661 (PMC4681533; doi:10.1371/journal.pone.0144661)

Supporting Information for

**Human excreta as a stable and important source of atmospheric ammonia in the megacity of Shanghai**

Yunhua Chang, Congrui Deng^*^, Anthony J. Dore, Guoshun Zhuang^*^

*To whom correspondence should be addressed. E-mail: [congruideng@fudan.edu.cn](mailto:congruideng@fudan.edu.cn) (CD) and [gzhuang@fudan.edu.cn](mailto:gzhuang@fudan.edu.cn) (GZ)

**S1 Fig. A schematic of the sampling in the exhaust of ceiling duct.** The right side is a schematic of the sampling in the exhaust of ceiling duct. The upper left is the wind speed & wind temperature data loger we used. The middle of the left is a Ogawa PSD in hand. The left bottom is a schematic diagram of the constrcture of Ogawa PSD.


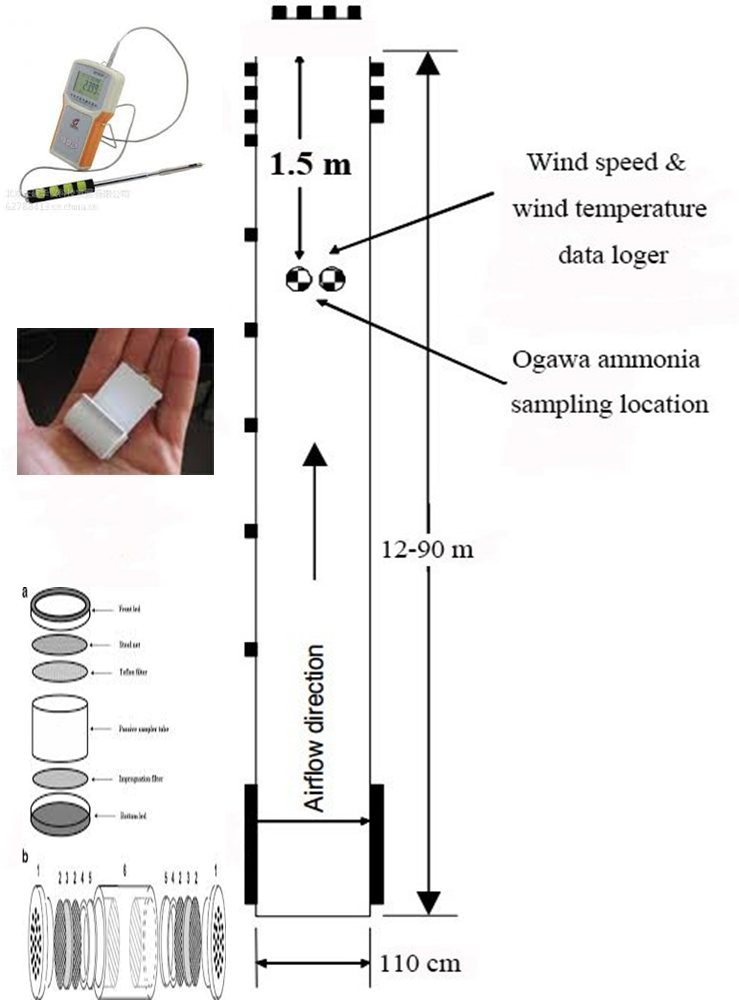

Supplement: S1 Fig — (DOCX) [file pone.0144661.s001.docx]
