# Supplementary material for: Human Excreta as a Stable and Important Source of Atmospheric Ammonia in the Megacity of Shanghai
Source: PLoS One. 2015 Dec 14;10(12):e0144661. doi: 10.1371/journal.pone.0144661 (PMC4681533; doi:10.1371/journal.pone.0144661)

Supporting Information for

**Human excreta as a stable and important source of atmospheric ammonia in the megacity of Shanghai**

Yunhua Chang, Congrui Deng^*^, Anthony J. Dore, Guoshun Zhuang^*^

*To whom correspondence should be addressed. E-mail: [congruideng@fudan.edu.cn](mailto:congruideng@fudan.edu.cn) (CD) and [gzhuang@fudan.edu.cn](mailto:gzhuang@fudan.edu.cn) (GZ)

**S2 Fig. Comparison of NH_3_ concentration results obtained with the Ogawa passive sampler device and an active monitor (MARGA) at Pudong supersite.**


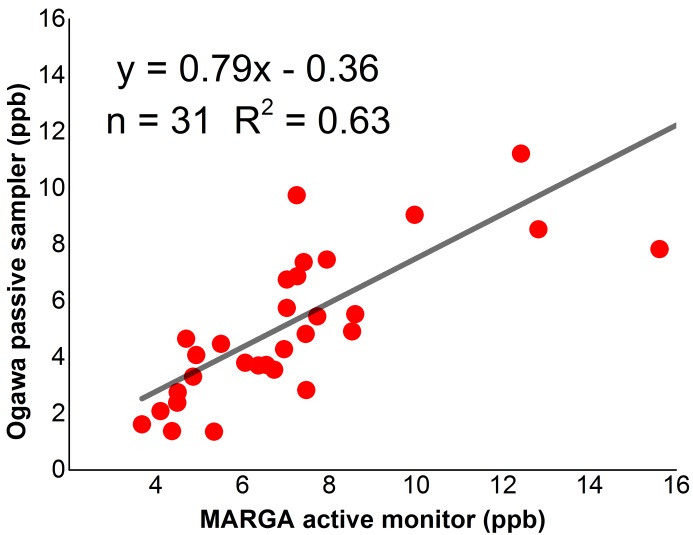

Supplement: S2 Fig — (DOCX) [file pone.0144661.s002.docx]
