# Supplementary material for: Human Excreta as a Stable and Important Source of Atmospheric Ammonia in the Megacity of Shanghai
Source: PLoS One. 2015 Dec 14;10(12):e0144661. doi: 10.1371/journal.pone.0144661 (PMC4681533; doi:10.1371/journal.pone.0144661)
Supplement: S1 Table — (DOCX) [file pone.0144661.s003.docx]

Supporting Information for

**Human excreta as a stable and important source of atmospheric ammonia in the megacity of Shanghai**

Yunhua Chang, Congrui Deng^*^, Anthony J. Dore, Guoshun Zhuang^*^

*To whom correspondence should be addressed. E-mail: [congruideng@fudan.edu.cn](mailto:congruideng@fudan.edu.cn) (CD) and [gzhuang@fudan.edu.cn](mailto:gzhuang@fudan.edu.cn) (GZ)

**S1 Table. Information about the thirteen buildings with six function types**

| **Building type** | **Building code** | **Property**  **owner** | **Number**  **of floor** | **Ceiling**  **duct code** | **Event** | **Basic description** |
| --- | --- | --- | --- | --- | --- | --- |
| Residential  Building^a^ | 17-A | Mr. Liu Jianguo | 17 | I |  | 16 families with 56 people^b^ |
|  | 17-B | Ms. Qu Ying | 17 | II |  | 16 families with 56 people^b^ |
|  | 11-A | Mr. Li Hua | 11 | III |  | 10 families with 35 people^b^ |
|  | 11-B | Mr. Huang Xin | 11 | IV |  | 10 families with 35 people^b^ |
|  | 8-A | Mr. Lian Zhu | 8 | V |  | 7 families with 21 people^b^ |
|  | 8-B | Mr. Dai Ming | 8 | VI |  | 7 families with 21 people^b^ |
| Hotel | HT | Mr. Bao Anlan | 5 | VII |  | 35 rooms and 4 ceiling  ducts in total^c^ |
|  |  |  |  | VIII |  |  |
| Office  Building | OB-1 | Mr. Wang Bo, the manager of Wanda Plaza | 21 | IX | Weekday | 24 000 m^2^ office space, fully occupied by people during working time^d^ |
|  |  |  |  |  | Weekend |  |
|  | OB-2 |  | 21 | X | Weekday | 24 000 m^2^ office space, fully occupied by people during working time^d, e^ |
|  |  |  |  |  | Weekend |  |
| Teaching  Building | TB | our own experimental site | 5 | XI (female) | Open | 35 classrooms and 2 ceiling  ducts collecting with a female toilet and a male toilet, respectively |
|  |  |  |  |  | Closed |  |
|  |  |  |  | XII  (male) | Open |  |
|  |  |  |  |  | Closed |  |
| Student  apartment | SA-1 | Ms. Sun Li, the manager of’ apartment | 7 | XIII |  | 7 rooms with 28 adult students^f^ |
|  | SA-2 |  | 7 | XIV |  | 7 rooms with 28 adult students^f^ |
| Community  culture  center | CC | Mr. Zhang Yuanming, the security guard of this building | 5 | XV |  | A entertainment complex  contains a small theater,  a dance hall, a gymnasium, four training rooms  and several office rooms^g^ |

a: each building has two indepent units, the building in current study is one of them. b: typical middle class family in Shanghai . Each family had a one child, most parents had a nine-to-five job. c: the hotel was ajacent to a key hospital in Shanghai, therefore, the occupancy rate always kept at a high level (＞80%). d: most occupants were the white-collar workers. e: many language tranning program were orgnized here. f: students at Fudan University. g: most people were the olders and the youngers.
