# Supplementary material for: Human Excreta as a Stable and Important Source of Atmospheric Ammonia in the Megacity of Shanghai
Source: PLoS One. 2015 Dec 14;10(12):e0144661. doi: 10.1371/journal.pone.0144661 (PMC4681533; doi:10.1371/journal.pone.0144661)
Supplement: S2 Table — (DOCX) [file pone.0144661.s004.docx]

Supporting Information for

**Human excreta as a stable and important source of atmospheric ammonia in the megacity of Shanghai**

Yunhua Chang, Congrui Deng^*^, Anthony J. Dore, Guoshun Zhuang^*^

*To whom correspondence should be addressed. E-mail: [congruideng@fudan.edu.cn](mailto:congruideng@fudan.edu.cn) (CD) and [gzhuang@fudan.edu.cn](mailto:gzhuang@fudan.edu.cn) (GZ)

**S2 Table. The NH_3_ concentrations, wind speed and emission factors of the six residential buildings and two student apartments**

| **Building type** | **Building code** | **Number of sampling** | **NH_3_ conc.**  **(μg m^-3^)** | **Average wind speed (m s^-1^)** | **Emission factor**  **(g NH_3_ capita^-1^ yr^-1^)** |
| --- | --- | --- | --- | --- | --- |
| Residential  Building | 17-A | 10 | 4149±1631 | 0.23 | 22.6±13.2 |
|  | 17-B | 11 | 3209±1581 | 0.20 | 16.5±10.2 |
|  | 11-A | 12 | 2679±2286 | 0.16 | 20.8±27.4 |
|  | 11-B | 13 | 3247±1118 | 0.18 | 22.1±13.7 |
|  | 8-A | 12 | 2662±1797 | 0.14 | 24.2±22.6 |
|  | 8-B | 13 | 2517±1414 | 0.15 | 25.3±22.9 |
| Student  apartment | SA-1 | 4 | 3116±1943 | 0.14 | 20.7±10.7 |
|  | SA-2 | 4 | 3397±1590 | 0.15 | 22.2±3.3 |
