# Supplementary material for: Human Excreta as a Stable and Important Source of Atmospheric Ammonia in the Megacity of Shanghai
Source: PLoS One. 2015 Dec 14;10(12):e0144661. doi: 10.1371/journal.pone.0144661 (PMC4681533; doi:10.1371/journal.pone.0144661)
Supplement: S1 Text — (DOCX) [file pone.0144661.s005.docx]

Supporting Information for

**Human excreta as a stable and important source of atmospheric ammonia in the megacity of Shanghai**

Yunhua Chang, Congrui Deng^*^, Anthony J. Dore, Guoshun Zhuang^*^

*To whom correspondence should be addressed. E-mail: [congruideng@fudan.edu.cn](mailto:congruideng@fudan.edu.cn) (CD) and [gzhuang@fudan.edu.cn](mailto:gzhuang@fudan.edu.cn) (GZ)

**S1 Text. The background information of the production and storage of human excreta in Shanghai and China.**

Firstly, according to China’s “Code for planning of urban environmental sanitation facilities” (GB 50337-2003), “fecal sewage front-end processing facility”, also known as septic tanks, are recommended in building construction for human excreta pre-treatment to avoid direct discharging of fecal sewage into the urban sewage pipe network. Although it is not mandatory to install septic tanks, to reduce the burden of wastewater treatment in sewage treatment plants, almost all cities in China have adopted the septic tank system (Cheng et al., 2010).

Secondly, the urban areas of China are facing the accumulation of human excreta and the poor management of septic tanks (Lu et al., 2008). According to *China urban construction statistical yearbook*, the collection and transportation amount of human excreta from urban areas has increased from 24220 kt in 1987 to 38050 kt in 2005 (the original statistical data can be obtained from [http://tongji.cnki.net/kns55/Navi/YearBook.aspx?id=N2014100078&floor=1###](http://tongji.cnki.net/kns55/Navi/YearBook.aspx?id=N2014100078&floor=1)).

We also noticed that in a few of China’s coastal cities, more and more human excreta has been treated by urban sewage treatment plants in recent years (Lei, 2011). In Shanghai, for example, the quantity of treated excrement has reached 637 kt (*China urban construction statistical yearbook*, 2014). Nevertheless, the treated human excreta have to be stored in the septic tanks first before treating. Besides, the collection and transportation amount of human excreta from septic tanks in 2013 (2220 kt) was much higher than that of treated excrement. Actually, the treatment level of human excreta in Shanghai has even not reached the level of Japan in 1975 (Lei, 2011). Therefore, there is still a long way for China to fully address the problem of human excreta in its urban areas.

As to the absolute number of septic tanks, superficially, the number of septic tanks in Shanghai in 2013 (43887) was slightly lower than the peak in 2002 (49220) (data obtained from *Shanghai Statistics Yearbook 2014*, available online: http://tongji.cnki.net/kns55/Navi/YearBook.aspx?id=N2014090171&floor=1###). However, the fact is that Shanghai has developed vertically at an unprecedented rate in the last decade. In this process, many low-rise houses with small-volume septic tanks in the city have been demolished and multiple septic tanks in a community were counted as one. According to the data from *Shanghai Urban Construction and Communications Commission*, Shanghai had 1463 buildings over 30 floors in 2013, while this figure is 212 in 2000. (<http://www.stats-sh.gov.cn/tjnj/nj14.htm?d1=2014tjnj/C1104.htm>). As a comparison, Shanghai rank as the first in terms of the number of buildings with height at least 100m in 2013 (the combined heights still lower than Hong Kong, https://en.wikipedia.org/wiki/List_of_cities_with_the_most_skyscrapers#cite_note-23).

**Reference**:

Cheng, H., H. Qiu, and M. Liu (2010), Review of setting septic tank in building outdoor drainage. *Fujian Architecture & Construction*, 129(150), 4-5. (in Chinses with English abstract)

Lu, Y., C. Tang, J. Chen, and Y. Sakura (2008), Impact of septic tank systems on local groundwater quality and water supply in the Pearl River Delta, China: case study, *Hydrological Process*, *22*(3), 443-450.

Lei, F. (2011), Research statues of black water treatment technology. *Guangdong Chemical Industry*, 38(4), 170-173. (in Chinses with English abstract)
